# Muscle-Specific SIRT1 Gain-of-Function Increases Slow-Twitch Fibers and Ameliorates Pathophysiology in a Mouse Model of Duchenne Muscular Dystrophy

**DOI:** 10.1371/journal.pgen.1004490

**Published:** 2014-07-17

**Authors:** Angeliki Chalkiadaki, Masaki Igarashi, Armiyaw Sebastian Nasamu, Jovana Knezevic, Leonard Guarente

**Affiliations:** Glenn Laboratory for the Science of Aging and Department of Biology, Massachusetts Institute of Technology, Cambridge, Massachusetts, United States of America; École Polytechnique Fédérale de Lausanne (EPFL), Switzerland

## Abstract

SIRT1 is a metabolic sensor and regulator in various mammalian tissues and functions to counteract metabolic and age-related diseases. Here we generated and analyzed mice that express SIRT1 at high levels specifically in skeletal muscle. We show that SIRT1 transgenic muscle exhibits a fiber shift from fast-to-slow twitch, increased levels of PGC-1α, markers of oxidative metabolism and mitochondrial biogenesis, and decreased expression of the atrophy gene program. To examine whether increased activity of SIRT1 protects from muscular dystrophy, a muscle degenerative disease, we crossed SIRT1 muscle transgenic mice to mdx mice, a genetic model of Duchenne muscular dystrophy. SIRT1 overexpression in muscle reverses the phenotype of mdx mice, as determined by histology, creatine kinase release into the blood, and endurance in treadmill exercise. In addition, SIRT1 overexpression also results in increased levels of utrophin, a functional analogue of dystrophin, as well as increased expression of PGC-1α targets and neuromuscular junction genes. Based on these findings, we suggest that pharmacological interventions that activate SIRT1 in skeletal muscle might offer a new approach for treating muscle diseases.

## Author Summary

Skeletal muscle has a central role in body posture, mobility and whole-body metabolism. SIRT1 is an enzyme expressed in skeletal muscle, as well as in most mammalian tissues, and has been shown to sense metabolic cues from the environment and mediate changes in these tissues, counteracting age and metabolic diseases. Here we generated and studied mice that express high levels of SIRT1 in skeletal muscle. We found that increased levels of SIRT1 in skeletal muscle led to gene expression changes similar to those that normally occur with endurance exercise. We also observed that SIRT1 overexpression counteracts muscle atrophy, a hallmark of aging muscle, and the muscle degenerative disease Duchenne muscular dystrophy (DMD). DMD is a debilitating disease caused by a mutation in the structural protein dystrophin. SIRT1 overexpression ameliorated the pathophysiology of DMD disease in a mouse model. Our results offer the hope that drugs that constitutively activate the enzymatic activity of SIRT1 might be used to cure muscle degenerative diseases.

## Introduction

Skeletal muscle has a central function in body stature and motility, as well as in energy storage, energy consumption, and whole-body metabolism. The various skeletal muscle groups consist of heterogeneous and specialized myofibers and are responsive and highly adaptable to contractile activity, nutrient availability, and hormones [1]. The different fiber types are characterized by specific biochemical, physiological, and metabolic parameters, which determine the function, size, metabolism, and fatigue resistance of each muscle group [2]. The myofibers are classified in two major types–the slow- and fast-twitch– with distinct contractile and metabolic properties. The slow-twitch myofibers contain mainly the type I myosin heavy chain isoform, are rich in mitochondria, and exhibit oxidative metabolism. The fast-twitch myofibers contain type IIa, IId/x, and IIb myosin heavy chain isoforms, are mainly glycolytic, perform quick contractions, and are required for movements involving strength and speed, but they are easily fatigued [3] [4] [5]. Most muscles consist of a mixture of fiber types, and the ratio is altered by exercise, or various systemic conditions, such as diabetes, cancer, and aging [2]. Calorie restriction, fasting, and exercise induce changes in skeletal muscle by transforming the myofibers from glycolytic to more oxidative forms rendering them more resistant to fatigue and atrophy [2] [6], [7]. Conversely, aging is associated with skeletal muscle atrophy, characterized by a progressive loss of oxidative fibers [6].

SIRT1, the mammalian orthologue of the yeast NAD^+^-dependent protein deacetylase Sir2 (silent information regulator 2), is expressed in various mammalian tissues, including skeletal muscle, and serves as a sensor and regulator of the energetic status of the cell, counteracting metabolic and age-related diseases [8], [9]. Under conditions of low glucose availability and increased energy demands, such as fasting, calorie restriction, and exercise, SIRT1 is induced in skeletal muscle and mediates mitochondrial biogenesis and fatty acid oxidation by deacetylating and regulating the activity of the transcriptional coactivator peroxisome-proliferator-activated receptor-gamma coactivator-1 α (PGC-1α) or forkhead box O (FOXO) transcription factors [10], [11], [12]. PGC-1α is a master regulator of mitochondrial gene expression [13], and transgenic overexpression has been shown to activate switching from fast-twitch to slow-twitch oxidative fibers in skeletal muscle, provide resistance to electrical stimulated fatigue [14] and protect from atrophy and metabolic disease during aging [15], [16] [17]. In aged muscle, NAD^+^ levels and SIRT1 activity decline, with a subsequent decrease in the expression of mitochondrial-encoded genes and mitochondrial homeostasis [18].

PGC-1α overexpression in skeletal muscle also ameliorates the phenotype of the X-linked recessive, muscle wasting disease Duchenne muscular dystrophy (DMD) [19]. DMD arises from a frameshift mutation in the gene dystrophin and leads to rapid degeneration of heart and skeletal muscle, causing disability and death by adolescence or young adulthood [20]. The dystrophic muscle is characterized by massive degeneration and necrosis of the damaged myofibers. It was observed that in DMD patients the fast-twitch fibers are more prone to damage, whereas the slow-twitch are relatively spared [21]. Active muscle regeneration–manifested by centrally localized nuclei– initially compensates for the degeneration but progressively the damaged muscle is replaced by connective and adipose tissues [22]. In normal muscle, dystrophin and the dystrophin-associated protein complex form a link between the intracellular actin based cytoskeleton and the extracellular matrix. Dystrophin is enriched at the junctions of muscle fibers and tendons and at the junctions of motor neurons with the muscle fibers (neuromuscular junction) [23], [24]. Lack of dystrophin protein leads to membrane destabilization and increased fragility, especially during intense contractile activity. PGC-1α stabilizes the weak cell membrane of the dystrophic myofiber by activating the neuromuscular junction (NMJ) gene program [19]. A strategy proposed to alleviate DMD involves the upregulation of utrophin [25], an autosomal ortholog of dystrophin, which partially compensates for dystrophin absence [26], [27], [28]. Because utrophin is expressed at higher levels in slow-twitch, oxidative fibers, it has been proposed that some of the effect of PGC-1α gain-of-function may be because it triggers conversion of fast-twitch to slow-twitch fibers [29].

In the current study, we explored the role of the metabolic regulator SIRT1 in skeletal muscle physiology under normal conditions and in the DMD model. We show that increased levels of SIRT1 in skeletal muscle drive a switch to slow-twitch fibers, reduce the muscle atrophy gene expression program, and ameliorate the DMD phenotype. In contrast, deletion of muscle SIRT1 exerts relatively minor phenotypes, suggestive of the presence of redundant mechanisms.

## Results

### Transgenic expression of SIRT1 in skeletal muscle induces a switch to slow-twitch oxidative fibers

**Figure 1:**
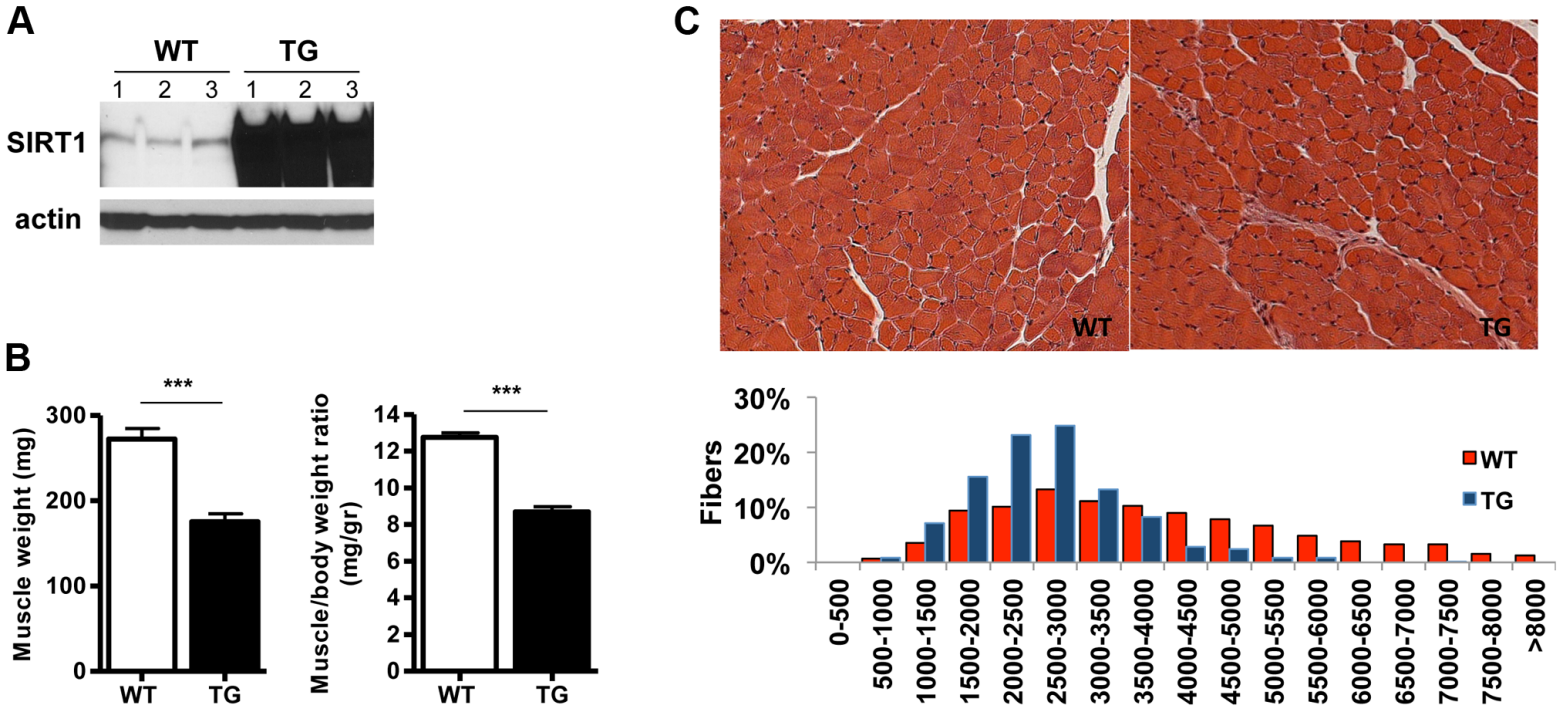
SIRT1 overexpression in skeletal muscle. (A) Western blot in tissue protein homogenates prepared from WT and transgenic gastrocnemius muscle (line 4140). All following experiments were performed using line Tg-4140, unless otherwise indicated. (B) Gastrocnemius muscle weight (of both hindlimbs) and muscle/body weight ratio of WT and Tg-4140 mice at 10 weeks of age (n = 7–10). (C) Representative H&E staining of gastrocnemius muscle from WT and transgenic mice and quantification of fiber size (arbitrary units) (700–1000 fibers/genotype, n = 3). Data are expressed as mean +/− s.e.m. ***p<0.001 by two-tailed unpaired Student's t test.

To study the role of SIRT1 in skeletal muscle physiology and in disease conditions, we generated skeletal muscle-specific SIRT1 overexpressing mice. The transgenic (Tg) mice were generated by injecting oocytes with a construct containing the cDNA of mouse *SIRT1* under the control of muscle creatine kinase promoter (*MCK*) [30]. We obtained three lines that all express higher than wild-type (WT) levels of SIRT1 in skeletal muscle: Tg-4140 (**Figure 1A** **and Figure S1A**), Tg-4145, and Tg-4311 (**Figure S1B and Figure S1C**). In most of the studies below we used the Tg-4140 line, unless otherwise indicated. The Tg mice were born to Mendelian ratios and their gross phenotype appeared normal. Whole-body weight of Tg-4140 mice was comparable to WT sibling controls, but muscle weight and muscle/body weight ratio were approximately 40% reduced compared to WT controls (**Figure 1B**). Histological analysis of WT and Tg-4140 gastrocnemius muscle by hematoxylin and eosin staining showed that Tg-4140 muscle had normal appearance (**Figure 1C**). We measured the cross-sectional area of WT and Tg-4140 fibers of gastrocnemius muscle and observed that Tg-4140 fibers were significantly smaller than WT (**Figure 1C**), which could explain why their muscles weigh less.

**Figure 2:**
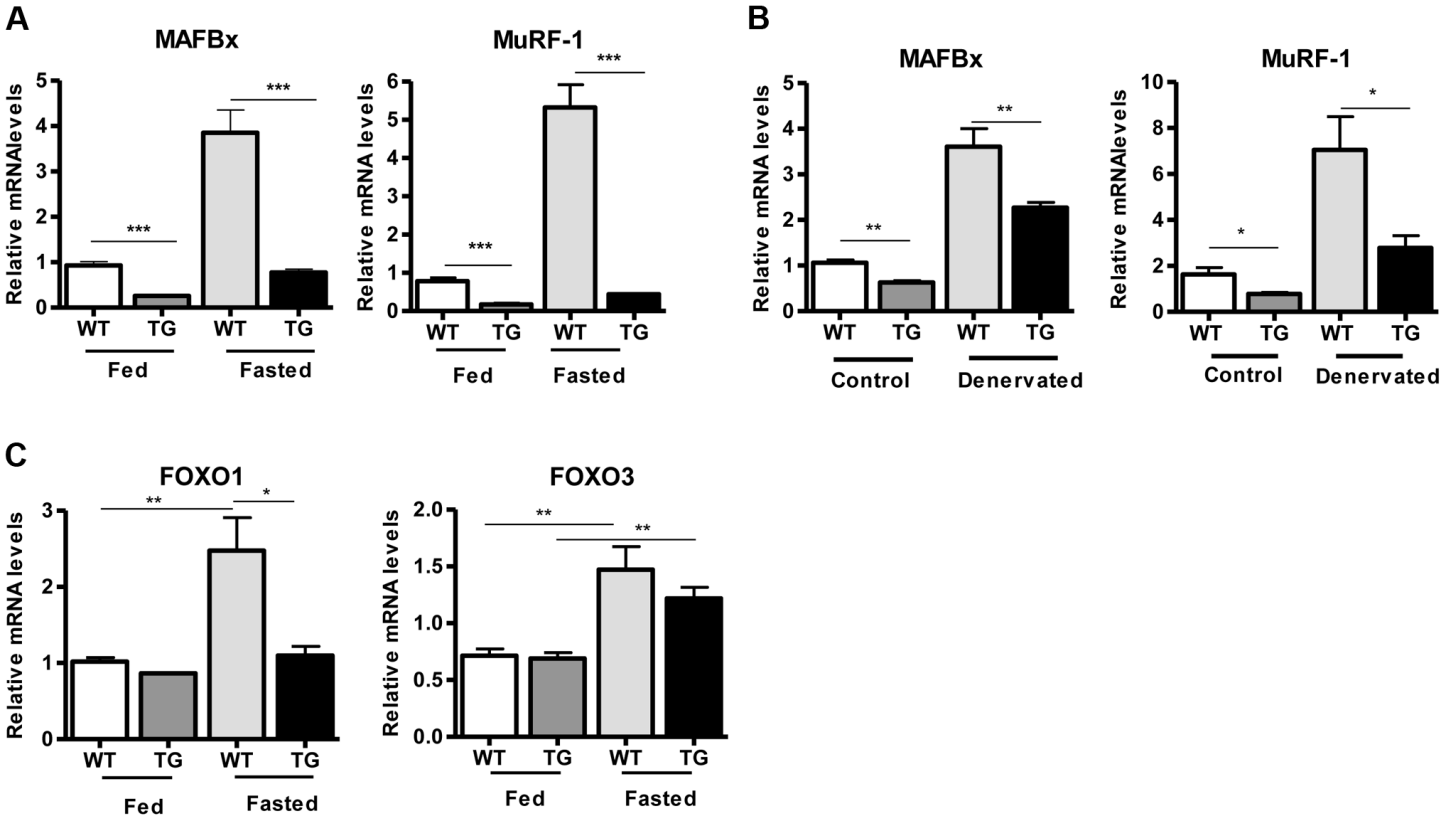
SIRT1 overexpression inhibits the expression of muscle atrophy genes. (A) Relative mRNA levels of *MAFBx* and *MuRF-1* atrophy genes in gastrocnemius muscle of WT and Tg-4140 mice fed or fasted for 24 hrs (n = 3–5). (B) Relative mRNA levels of *MAFBx* and *MuRF-1* atrophy genes in gastrocnemius muscle of WT and Tg-4140 mice, which underwent mock surgery (control) or were denervated for 3 days (n = 3–5). (C) Relative mRNA levels of *FOXO1* and *FOXO3* transcription factors in gastrocnemius muscle of WT and Tg-4140 mice fed or fasted for 24 hrs (n = 3–5). Data are expressed as mean +/− s.e.m. *p<0.05, **p<0.01, ***p<0.001 by two-tailed unpaired Student's t test.

To examine whether SIRT1 overexpression in skeletal muscle activates the muscle wasting gene program, resulting in increased proteasomal degradation and therefore smaller muscles, we measured the expression levels of atrophy genes by quantitative RT-PCRs, under basal and atrophy-inducing conditions. We observed that under basal conditions the expression levels of the two E3 ubiquitin ligases, hallmarks of skeletal muscle atrophy, *MAFBx* and *MuRF1*, were actually reduced in Tg-4140 muscles compared to WT (**Figure 2A**). To induce muscle atrophy we subjected WT and Tg-4140 mice to either 24 hr fasting (**Figure 2A**) or 3 days of disuse induced by denervation after sectioning the sciatic nerve (**Figure 2B**). We verified that *MAFBx* and *MuRF1* genes were strongly induced in WT muscles under both conditions of atrophy (**Figure 2A and 2B**), as previously described [31], [32]. However, the induction of the atrophy markers in Tg-4140 was significantly less compared to WT (**Figure 2A and 2B**). It was previously shown that the FOXO transcription factors are induced in fasting atrophy and are necessary for the atrophy gene program in skeletal muscle [33], [34]. So we tested whether SIRT1 overexpression affects the induction of FOXO transcription factors after fasting, and we observed that *FOXO1* was not induced in Tg-4140 muscle compared to WT after 24 hr fasting, whereas the induction of *FOXO3* mRNA was only weakly affected (**Figure 2C**). In conclusion, SIRT1 overexpression does not induce muscle wasting; on the contrary, it counteracts the atrophy gene program.

**Figure 3:**
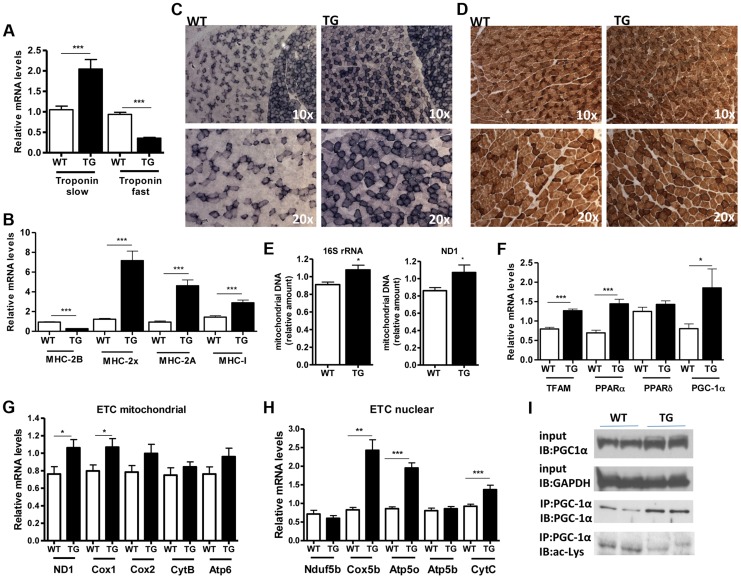
SIRT1 overexpression drives fast-to-slow fiber type switch. (A) Relative mRNA levels of troponin slow and troponin fast genes in gastrocnemius muscle of WT and Tg-4140 mice (8–10 weeks old, n = 3–5). (B) Relative mRNA levels of myosin heavy chain 2B, 2x, 2A, and I in gastrocnemius muscle of WT and Tg-4140 mice (n = 3–5). (C) Representative SDH activity staining of cross-sections of gastrocnemius/soleus (upper panels) and gastrocnemius (lower panel) muscle of WT and Tg-4140 mice. Quantitation is shown in **Figure S2A**. (D) Representative COX activity staining of cross-sections of gastrocnemius muscle of WT and Tg-4140 mice. Quantitation is shown in **Figure S2B**. (E) Relative mitochondrial DNA of indicated genes normalized to actin (10–12 weeks old, n = 4). (F) Relative mRNA levels of *TFAM*, *PPARα*, *PPARδ*, and *PGC-1α* in gastrocnemius muscle of WT and Tg-4140 mice (n = 3–5). (G) Relative mRNA levels of mitochondrial-expressed electron transport chain (ETC) genes in gastrocnemius muscle of WT and Tg-4140 mice (10–12 weeks old, n = 3–5). (H) Relative mRNA levels of nuclear-expressed electron transport chain (ETC) genes in gastrocnemius muscle of WT and Tg-4140 mice (10–12 weeks old, n = 3–5). (I) Lysine acetylation levels of PGC-1α (IB: ac-Lys) in protein extracts prepared from gastrocnemius muscle of WT and Tg-4140 and immunoprecipitated by PGC-1α specific antibodies (IP: PGC-1α). Data are expressed as mean +/− s.e.m. *p<0.05, **p<0.01, ***p<0.001 by two-tailed unpaired Student's t test.

A role of SIRT1 in skeletal muscle physiology was suggested by the induction of its activity during exercise and calorie restriction [10], [11], [35]. Both conditions drive fiber type switch, mitochondrial biogenesis, and more oxidative metabolism [3]. So overexpression of SIRT1 could alter the composition of fibers, inducing the formation of more oxidative, slow-twitch fibers, which are smaller in size. To test this hypothesis, we measured the expression levels of various fiber type markers by quantitative RT-PCRs in gastrocnemius muscle of WT and Tg-4140 mice. We observed a switch towards more oxidative slow-twitch type in Tg-4140 muscle as manifested by an increase in markers of slow-twitch and more oxidative fibers (troponin slow) and a concomitant decrease in fast-twitch and more glycolytic myofibers (troponin fast) (**Figure 3A**). In addition, we measured the relative gene expression levels of myosin heavy chain isoforms, and we observed an increase in *MHC-I* and in the isoforms of *MHC-2* (*2x* and *2A*) associated with slow-twitch oxidative fibers [36] and a concomitant reduction in *MHC-2B* isoform associated with fast-twitch fibers [36] (**Figure 3B**). Histological analyses of gastrocnemius/soleus muscle enzymatically stained for the mitochondrial enzyme succinate dehydrogenase (SDH) (**Figure 3C** **and Figure S2A**) and cytochrome oxidase (COX) (**Figure 3D** **and Figure S2B**), as well as measurements of mitochondrial DNA content (**Figure 3E**), verified that transgenic muscle contains more oxidative fibers and increased mitochondrial activity. In addition, myosin ATPase activity staining showed a 50% increase in type I fibers in transgenic gastrocnemius muscle (**Figure S2C**). Consistent with the fiber type switch and increased mitochondrial content and activity, genes encoding transcription factors associated with increased mitochondrial gene expression, such as *PGC-1α*, *TFAM*, and *PPARα*, mitochondrial proteins such as cytochrome c and some of the electron transport chain proteins were upregulated in Tg-4140 muscle (**Figure 3F, 3G, and 3H**), as well as in muscle of Tg-4311 and Tg-4145 mice (**Figure S1D, S1E, S1F and S1G**). PGC-1α is central in the regulation of mitochondrial biogenesis in skeletal muscle, and SIRT1 is known to activate its transcriptional activity by deacetylation [12], [37]. We examined whether PGC-1α protein is differentially acetylated in Tg-4140 muscle by immunoprecipitation followed by western blot (**Figure 3I**). First, we observed that PGC-1α protein is induced in Tg-4140 muscle (**Figure 3I**), consistent with the increased RNA levels we observed, and a previously reported positive autoregulation of *PGC-1α* promoter by PGC-1α protein [38]. Immunoprecipitation of PGC-1α from WT and transgenic muscle followed by western blot with anti-acetyl lysine antibodies showed that the levels of acetylation of PGC-1α in transgenic muscle are reduced, in agreement with the known role of SIRT1 in PGC-1α acetylation status and activity (**Figure 3I**). From these data we concluded that SIRT1 overexpression results in fiber type switch towards more oxidative metabolism and increased mitochondrial activity.

**Figure 4:**
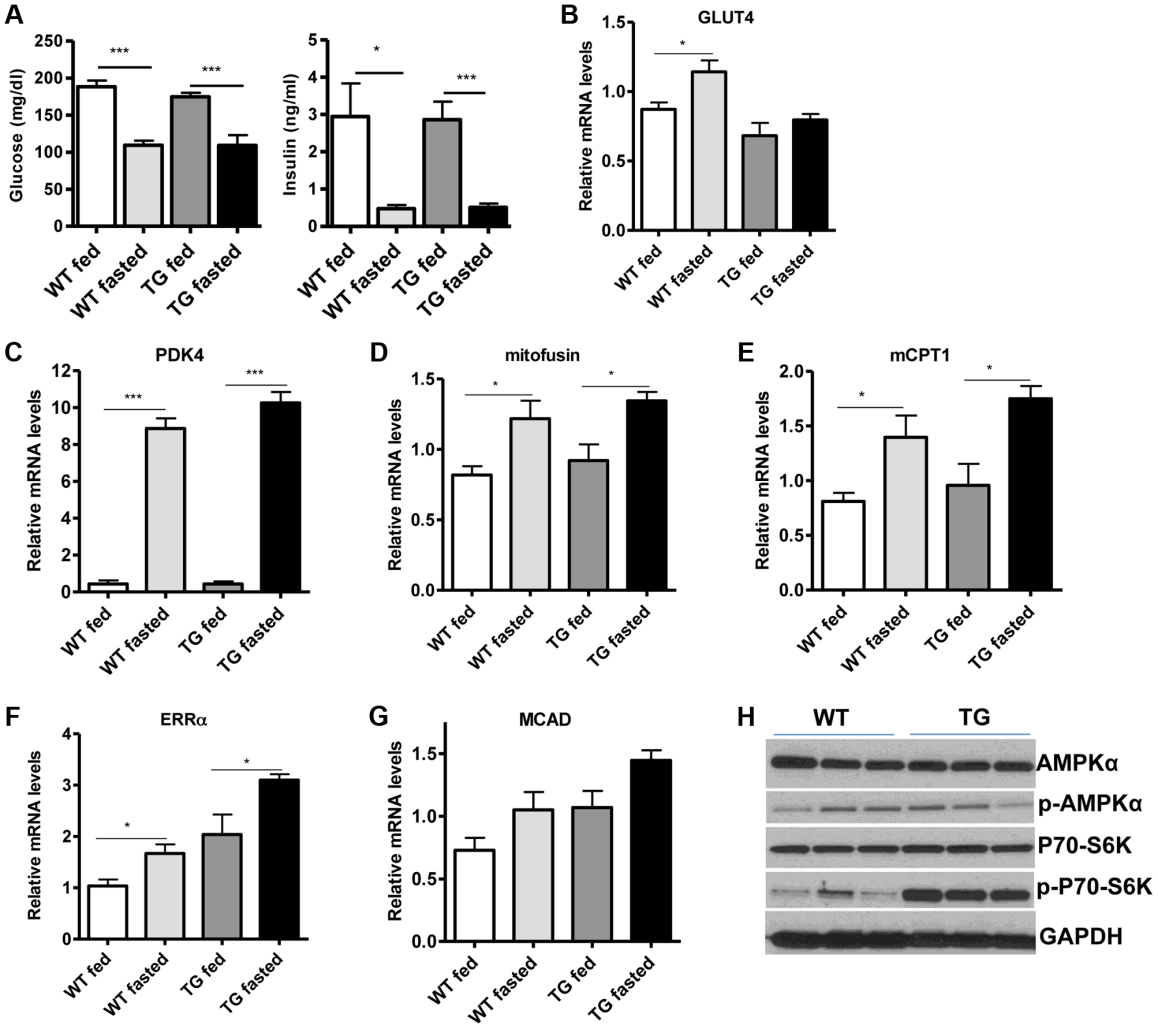
SIRT1 overexpression in skeletal muscle does not affect the fasting response. (A) Blood glucose and plasma insulin levels in fed or after overnight fasting of WT and Tg-4140 mice (8–10 weeks old, n = 6–10). (B–G) Relative mRNA levels of *GLUT4*, *PDK4*, mitofusin, *mCPT1*, *ERRα*, and *MCAD* genes in gastrocnemius muscle of fed and fasted WT and Tg-4140 (10–12 weeks old, n = 4). (H) Western blot analyses in gastrocnemius muscle protein extracts prepared from gastrocnemius muscle of WT and Tg-4140 (10–12 weeks old). Data are expressed as mean +/− s.e.m. *p<0.05, **p<0.01, ***p<0.001 by two-tailed unpaired Student's t test.

Skeletal muscle consumes large amounts of energy in the body by glucose uptake. Because SIRT1 has a central role in energy sensing and metabolic regulation, we tested whether SIRT1 overexpression in the muscle affects whole-body glucose homeostasis and response to fasting. We found that fed and fasting glucose and insulin levels in the blood of Tg-4140 mice were comparable to WT controls (**Figure 4A**). In addition, we measured the levels of genes that are known to respond to fasting and we found that Tg-4140 muscle adapts to fasting as efficiently as WT (**Figure 4B, 4C, 4D, 4E, 4F, and 4G**). Previous evidence showed that there is an interdependence of the energy sensor AMP-activated protein kinase (AMPK) and SIRT1 in skeletal muscle, with AMPK upregulating the NAD^+^ levels and SIRT1 activity upon fasting or exercise [10], [11], [39]. We examined whether overexpression of SIRT1 alters AMPK activity under basal fed conditions by western blot analysis using phospho-specific antibodies, and we observed that the phosphorylation status of the catalytic subunit AMPKα at Thr172, which is required for AMPK activation, remains unaltered in Tg-4140. The mammalian target of rapamycin (mTOR) is also a central energy sensor and functions to balance nutrient availability and cell growth [40]. In Tg-4140 muscle, the phosphorylation of the downstream target of mTOR pathway p70 S6 kinase (p70-S6K) at Thr389 is increased, suggesting that mTOR activity is induced (**Figure 4H**). Future studies will further investigate the mechanism by which SIRT1 overexpression leads to activation of mTOR pathway. In conclusion, overexpression of SIRT1 in skeletal muscle drives the formation of more oxidative fibers but cannot alter basal whole-body glucose homeostasis and the response to fasting.

### Loss of SIRT1 from skeletal muscle does not affect oxidative metabolism or myofiber composition

**Figure 5:**
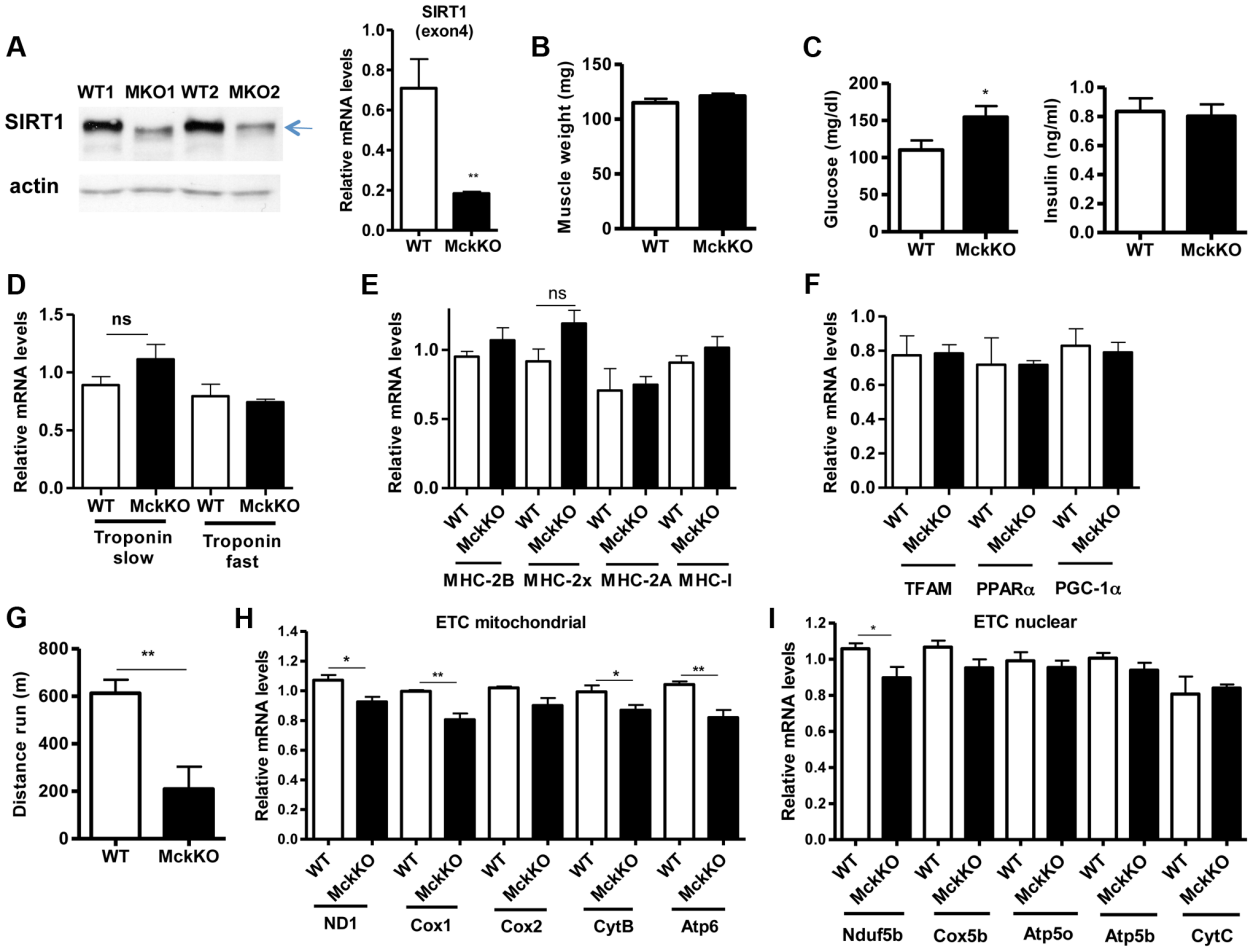
Loss of SIRT1 from skeletal muscle does not affect fiber type composition but reduces the capacity for endurance exercise. (A) Western blot in tissue protein homogenates prepared from WT and muscle-specific SIRT1 knockout (MckKO) gastrocnemius muscle. The arrow shows the faster migrating SIRT1 protein lacking the catalytic domain (ΔExon4). Relative RNA levels of *SIRT1* transcript using SIRT1 exon 4 specific primers. (B) Gastrocnemius muscle weight (of one hindlimb) of WT and MckKO mice at 10 weeks of age (n = 7–10). (C) Blood glucose and plasma insulin levels in WT and MckKO mice after overnight fasting (8–10 weeks old, n = 6–10). (D) Relative mRNA levels of troponin slow and troponin fast genes in gastrocnemius muscle of WT and MckKO mice (8–10 weeks old, n = 3–5). (E) Relative mRNA levels of myosin heavy chain 2B, 2x, 2A, and I in gastrocnemius muscle of WT and MckKO mice (n = 3–5). (F) Relative mRNA levels of *TFAM*, *PPARα*, and *PGC-1α* in gastrocnemius muscle of WT and MckKO mice (n = 3–5). (G) Distance run in treadmill exercise by WT and MckKO mice (12–14 weeks old, n = 10–12). (H) Relative expression levels of mitochondrial-expressed electron transport chain (ETC) genes (10–12 weeks old, n = 4). (I) Relative expression levels of nuclear-expressed ETC genes (10–12 weeks old, n = 4). Data are expressed as mean +/− s.e.m. *p<0.05, **p<0.01, ***p<0.001 by two-tailed unpaired Student's t test.

To examine whether SIRT1 activity is necessary for oxidative metabolism and fiber type composition in skeletal muscle, we generated muscle-specific *SIRT1* knockout (MckKO) mice, by crossing mice expressing the cre recombinase under the control of *MCK* promoter [41] to mice carrying the floxed *SIRT1* allele [42]. The loxP sites flank the exon 4 of *SIRT1* gene, which corresponds to the catalytic domain of the enzyme, so cre-mediated excision results in a smaller SIRT1 protein that lacks its enzymatic activity (**Figure 5A**). The appearance and weight of MckKO muscle were comparable to WT controls (**Figure 5B**). Blood glucose levels were slightly elevated in MckKO mice, but insulin levels were normal (**Figure 5C**). Expression levels of troponin isoforms (**Figure 5D**), myosin heavy chain isoforms (**Figure 5E**), and mitochondrial transcription factors (**Figure 5F**) did not differ between WT and MckKO muscles by quantitative RT-PCRs. Thus SIRT1 loss from skeletal muscle did not affect the expression levels of mitochondrial transcription factors or myofiber type composition, which is in agreement with recently published papers [43], [44]. We challenged MckKO and WT sibling controls by forced treadmill exercise to exhaustion. We employed a mild running protocol and we observed no difference between WT and MckKO mice. However, when we used a more intense exercise protocol we observed that MckKO mice were exhausted faster and ran shorter distance compared to WT mice (**Figure 5G**). A possible explanation for intolerance in exercise is defective mitochondrial function. To further investigate this possibility, we measured the expression levels of mitochondrial- and nuclear-expressed electron transport chain (ETC) genes, and we found that the mitochondrial- but not the nuclear- expressed ETC genes are slightly but significantly reduced in MckKO muscle (**Figure 5H and 5I**). Our observations are consistent with recent findings showing that SIRT1 regulates mitochondrial-encoded ETC genes [18] and suggest that MckKO mice are more sensitive to fatigue after acute exercise, possibly because of reduced expression of mitochondrial-expressed ETC genes.

Prompted by our observations that SIRT1 overexpression results in lower expression levels of atrophy genes, under basal and atrophy inducing conditions, we examined the levels of these genes in gastrocnemius muscle from MckKO mice. Quantitative RT-PCR analyses showed that absence of SIRT1 did not affect the levels of *MAFBx* and *MuRF1* under basal or atrophy-inducing conditions (**Figure S3A and S3B**). In summary, deletion of SIRT1 in muscle exerts only a subtle phenotype under the conditions tested.

### SIRT1 transgenic mice express high levels of utrophin and neuromuscular junction genes in skeletal muscle

**Figure 6:**
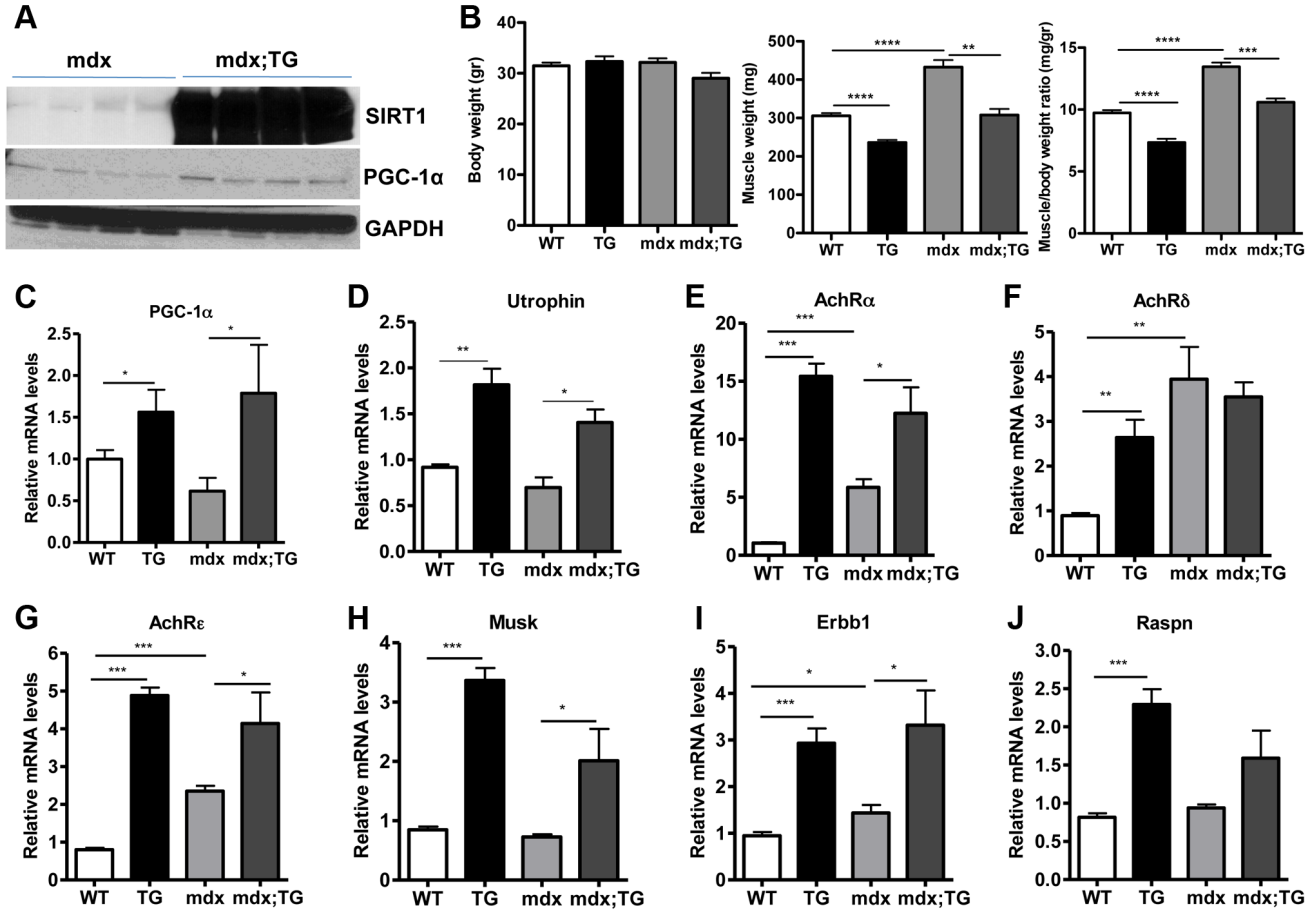
SIRT1 overexpression in skeletal muscle of WT and mdx mice induces the expression of neuromuscular junction genes and utrophin. (A) Western blot in tissue protein homogenates prepared from gastrocnemius muscles of mdx and mdx mice crossed to Tg-4140 (mdx;TG) (B) Body weight, gastrocnemius muscle weight (of both hindlimbs) and muscle/body weight ratio of WT, Tg-4140, mdx, and mdx;Tg-4140 mice at 10–14 weeks of age (n = 5–10). (C–J) Relative mRNA levels of *PGC-1α*, utrophin, acetylcholine receptor (AchR) subunits α, δ, ε, and *Musk*, *Erbb1*, and *Raspn* in gastrocnemius muscle of WT, Tg-4140, mdx, and mdx;Tg-4140 mice (n = 3–5). Data are expressed as mean +/− s.e.m. *p<0.05, ** p<0.01, ***p<0.001 by two-tailed unpaired Student's t test.

Several lines of evidence led us to hypothesize that SIRT1 overexpression in muscle could counteract the muscular degenerative disease DMD. For example, it is known that slow-twitch, oxidative fibers are less prone to degeneration compared to fast-twitch glycolytic fibers [21]. As SIRT1 transgenic muscles contain more slow-twitch fibers (**Figure 3**), transgenic mice might be protected from muscle degeneration. In addition, slow-twitch fibers express higher levels of utrophin, the functional analogue of dystrophin that could compensate to some extent for dystrophin's loss in DMD. In addition, SIRT1 overexpression leads to increased levels of PGC-1α, known to protect against DMD [19]. We thus crossed SIRT1 Tg-4140 and Tg-4145 mice to mdx mouse model [45] (**Figure 6A**), which is a severe model of Duchenne muscular dystrophy. We measured body and muscle weights of all four groups (WT, Tg, mdx, and mdx;Tg). Our results showed that SIRT1 overexpression in mdx muscle reverses the characteristic muscle hypertrophy of dystrophic muscle caused by vigorous regeneration following muscle damage and necrosis (**Figure 6B**). We tested whether overexpression of SIRT1 in the mdx mouse also leads to increased levels of PGC-1α, as in the WT genetic background, and we observed that both RNA (**Figure 6C**) and protein levels (**Figure 6A**) are increased. To verify that SIRT1 overexpression affects the expression levels of utrophin, we performed quantitative RT-PCR analysis and we observed that utrophin levels are increased more than 2-fold in gastrocnemius muscle of Tg-4140, 1.5 fold in mdx;Tg-4140 (**Figure 6D**), and 1.5 fold in Tg-4145 (**Figure S4A**) mice.

PGC-1α, a bona-fide target of SIRT1, stimulates the neuromuscular gene program (NMJ), as a coactivator of GABP transcription factor, and counteracts the abnormalities of NMJ morphology in a muscular dystrophy model [19]. Thus, we examined whether SIRT1 overexpression in skeletal muscle also leads to increased levels of NMJ genes, and we observed that transcripts of the acetylcholine receptor subunits, and components of the acetylcholine signaling, were all expressed at dramatically higher levels in Tg-4140 and mdx;Tg-4140 muscles compared to WT and mdx muscles, respectively (**Figure 6E, 6F, 6G, 6H, 6I, and 6J**). Intriguingly, mdx mice themselves showed a modest upregulation of the three *AchR* genes and *Erbb1* gene– but not the other NMJ genes– compared to WT, in agreement to previous observations [46], possibly representing a compensatory response of acetylcholine receptors in mdx mice.

### SIRT1 overexpression in skeletal muscle alleviates the phenotype of muscular dystrophy

**Figure 7:**
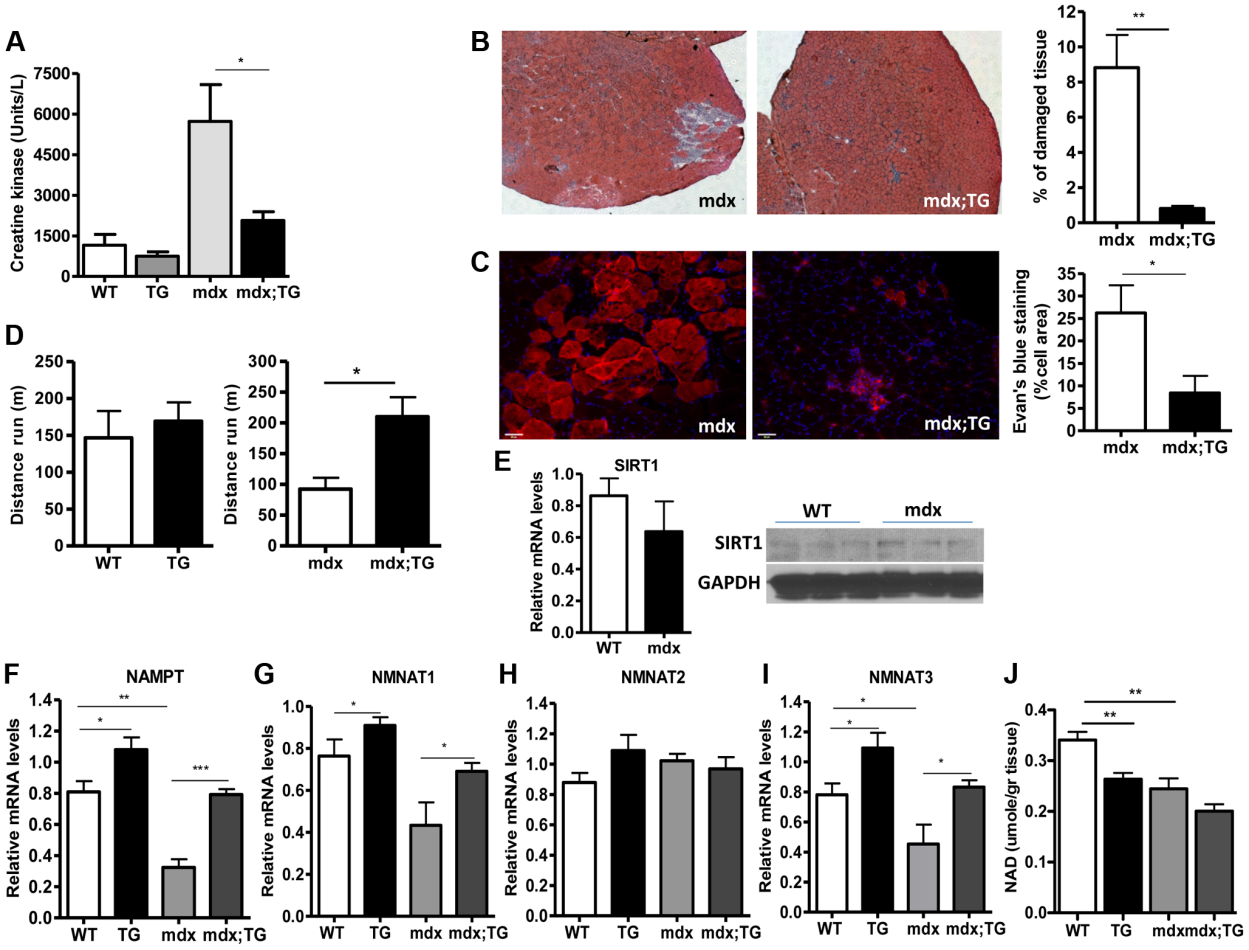
SIRT1 overexpression in skeletal muscle alleviates the muscular dystrophic phenotype of mdx mouse. (A) Serum creatine kinase activity in WT, Tg-4140, mdx, mdx;Tg-4140 mice (8–10 weeks old, n = 10). (B) Representative H&E staining of gastrocnemius muscle from mdx and mdx;Tg-4140 mice and quantification of damaged area (10 sections/genotype, n = 3). (C) Representative Evans Blue staining of gastrocnemius muscle from mdx and mdx;Tg-4140 mice and quantification of staining area (10 sections/genotype, n = 3). (D) Distance run in treadmill exercise by WT and Tg-4140 mice, and mdx and mdx;Tg-4140 mice (12–14 weeks old, n = 10–12). (E) Relative mRNA levels of *SIRT1* in gastrocnemius muscle of WT and mdx mice (10–12 weeks old, n = 4). Western blot analysis of protein extracts prepared from gastrocnemius muscle of WT and mdx mice. (F–I) Relative mRNA levels of *NAMPT*, *NMNAT1*, *NMNAT2*, and *NMNAT3* in gastrocnemius muscle from WT, Tg-4140, mdx and mdx;Tg-4140 mice (n = 3–5). (J) NAD^+^ levels in gastrocnemius muscle from WT, Tg-4140, mdx and mdx;Tg-4140 mice (n = 3–5). Data are expressed as mean +/− s.e.m. *p<0.05, **p<0.01, ***p<0.001 by two-tailed unpaired Student's t test.

Our observations that SIRT1 transgenic muscle consists of higher percentage of slow-twitch oxidative myofibers and express high levels of utrophin and NMJ genes prompted us to test whether SIRT1 overexpression protects from muscular dystrophy. We first measured the levels of serum creatine kinase (CK), a hallmark of damaged muscles, in young adult WT, Tg, mdx, and mdx/Tg mice. As expected, we observed a large increase in CK activity in mdx mice compared to WT mice. Critically, we found that overexpression of SIRT1 in Tg-4140 and Tg-4145 mice resulted in a ∼50% reduction of CK enzymatic activity in the mdx genetic background (**Figure 7A** **and Figure S4B**). Another hallmark of dystrophic muscles is the large percentage of myofibers with centrally localized nuclei, indicative of regenerating tissue after damage, and large areas of infiltrating macrophages and fibroblasts. We compared the percentage of non-muscle cells infiltrating the gastrocnemius muscle in mdx;Tg-4140 muscle with mdx muscle, by hematoxylin and eosin staining. The mdx muscle contains approximately 8% of fibrotic tissue, whereas SIRT1 overexpression in mdx;Tg-4140 mice resulted in a reduction of fibrosis to about 1% (**Figure 7B**). To further assess the extent of damaged myofibers, we injected intraperitoneally Evans Blue dye, which only penetrates and stains damaged cells. Evans Blue dye stained ∼25% of myofibers of mdx mice, whereas overexpression of SIRT1 in mdx;Tg-4140 mice reduced the stained myofibers to ∼8% (**Figure 7C**).

Individuals with DMD have weak muscles, which are quickly exhausted. Similarly, the mdx mice when forced to run are fatigued much faster compared to WT controls, as the mdx fibers are more vulnerable [47]. To assess the effects of SIRT1 overexpression on physical performance of mdx mice, we challenged mdx and mdx;Tg-4140 mice with treadmill exercise to exhaustion. While the mdx mice ran on average 100 m, the mdx;Tg-4140 mice ran significantly more, ∼250 m, a distance comparable to WT mice (**Figure 7D**). Overall, these results suggest that SIRT1 overexpression in skeletal muscle dramatically improves the pathophysiology of the muscular dystrophic phenotype.

Next, we asked whether SIRT1 levels decline in mdx muscle. We assessed SIRT1 RNA and protein levels, and we observed that SIRT1 is expressed in WT and mdx muscle at comparable levels (**Figure 7E**). Since NAD^+^ is a rate limiting co-substrate for SIRT1, it is possible that NAD^+^ levels are altered in mdx muscle affecting SIRT1 activity. We assessed the expression levels of the enzymes in the NAD^+^ biosynthetic pathway and we observed that *NAMPT* (mitochondrial), *NMNAT1* (nuclear), and *NMNAT3* (mitochondrial), are reduced in mdx muscle, suggesting that NAD^+^ levels are also reduced (**Figure 7F, 7G, 7I**). We did not observe any significant difference in *NMNAT2* (Golgi) (**Figure 7H**). We directly measured NAD^+^ levels in WT and mdx muscle and we observed that the dystrophic muscle has lower levels of NAD^+^, in agreement to the reduced expression levels of NAD^+^ biosynthetic enzymes (**Figure 7J**). Interestingly, we observed that SIRT1 overexpression induces the expression of *NAMPT*, *NMNAT1*, and *NMNAT3* in WT and mdx genetic background. Conversely, overexpression of SIRT1 results in moderate reduction of cellular NAD^+^ levels in WT genetic background, probably caused by increased consumption. The increase in NAD^+^ synthetic enzymes may be a compensatory mechanism for increased NAD^+^ consumption in transgenic mice. Overall our results show that the expression levels of NAD^+^ biosynthetic enzymes and NAD^+^ concentration are reduced in dystrophic muscle, suggesting that the activity of NAD^+^-dependent enzymes, such as SIRT1, is downregulated. In SIRT1 transgenic mice, the reduced NAD^+^ likely corresponds to increased SIRT1 activity and protein levels.

Finally, we examined whether absence of SIRT1 worsened the dystrophic phenotype of mdx mice by crossing them to mice lacking SIRT1 from muscle (MckKO). We found that CK activity in mdx;MckKO mice was similar to mdx sibling controls (**Figure S4C**), as was the endurance of these mice when subjected to treadmill exercise to exhaustion (**Figure S4D**). In agreement with these results, SIRT1 loss does not affect the expression levels of neuromuscular junction genes in WT or mdx mice (**Figure S4E and S4F**). These results are consistent with the observations that knocking out SIRT1 in WT genetic background exerts only a subtle phenotype on mice fed normal chow diet [39], [43], [44], [48].

## Discussion

SIRT1 is an important metabolic regulator in mammals; it is induced under energy-limiting conditions in various tissues, including skeletal muscle, and deacetylates and regulates the activity of target proteins [8], [49]. Here we generated SIRT1 muscle overexpressing and knockout mice to study the role of SIRT1 in skeletal muscle *in vivo*. Our data show that SIRT1 overexpression results in a fast-to-slow fiber type switch that translates into an increase in oxidative fibers. These observations correlate with higher expression of PGC-1α in SIRT1 Tg muscle and are in agreement with previous observations made with PGC-1α transgenic mice. A number of studies have shown that SIRT1 deacetylates and positively regulates PGC-1α in cell culture systems and under fasting or exercise conditions *in vivo* [10], [12], [37]. Overexpression of PGC-1α in skeletal muscle regulates mitochondrial biogenesis, activates oxidative metabolism, drives fast-to-slow fiber switch [14] and protects from muscular atrophy and muscular dystrophy [15], [19]. Moreover, muscle specific PGC-1α KO mice showed a shift from oxidative type I and IIa toward type IIx and IIb muscle fibers, reduced endurance capacity in treadmill exercise and increased muscle damage [50], [51].

However, recent papers showed that muscle PGC-1α is dispensable for voluntary exercise-induced mitochondrial biogenesis [52], [53], pointing that there are other unidentified factors and pathways that regulate mitochondrial biogenesis during exercise. Similarly, our data regarding SIRT1 loss-of-function from skeletal muscle show that SIRT1 is dispensable for the expression of mitochondrial genes, fiber type composition in sedentary animals, changes in fiber composition after voluntary exercise, and atrophy, suggesting that redundant mechanisms regulate these pathways in skeletal muscle. However, we did find that loss of muscle SIRT1 resulted in a modest increase in blood glucose levels and less endurance of mice to treadmill exercise.

Importantly, knocking out SIRT1 in muscle was shown to affect the response to calorie restriction [48], a condition known to increase sirtuin protein levels and activity in muscle [35], [54]. Thus the properties of SIRT1 transgenic mice may mimic calorie-restricted mice, and are consistent with the fact that they display an increase in mitochondrial biogenesis and of slow-twitch oxidative fibers. Most dramatically, our transgenic mice are protected against tissue degeneration in a model of DMD, as detailed below.

DMD is a debilitating disease affecting 1 in 3,500 boys worldwide resulting in muscle degeneration and death. To date, effective pharmacological treatment for DMD is not available. In this study we showed that increased levels of SIRT1 in skeletal muscle of the mouse model of DMD, mdx, ameliorates the disease phenotype and improves muscle physiology and function. It was previously shown that the transcriptional coactivator PGC-1α improves the disease phenotype by regulating the NMJ genes, inducing utrophin expression, and increasing the percentage of oxidative fibers [19], [29], which are more resistant to degeneration. SIRT1 muscle-specific overexpressing mice phenocopy the PGC-1α transgenic mouse: they express high levels of markers of slow-twitch myofibers, high levels of utrophin, and high levels of NMJ genes. Importantly, SIRT1 reverses the phenotype of the mdx mouse as evidenced by intact muscles, less creatine kinase activity in the blood, and better performance in treadmill exercise. Interestingly, we observed that the expression levels of NAD^+^ biosynthetic enzymes and NAD^+^ concentration are reduced in dystrophic muscle, suggesting that the activity of NAD^+^-dependent enzymes, such as SIRT1, is downregulated. The reduction in NAD^+^ levels in SIRT1 Tg mice likely reflects increased SIRT1 activity (which degrades NAD^+^) in these mice, and is consistent with the suppression of phenotypes in mdx SIRT1 overexpressing mice.

Since SIRT1 can be activated by small molecules [55], our results offer promise for pharmacological interventions that can activate SIRT1 and alleviate the dystrophic phenotype in patients. In support of this notion, a recently published paper showed that resveratrol, a first generation SIRT1 activator [56], improved some of the dystrophic phenotypes such as myofiber loss and fibrosis but did not have any effects on the high levels of CK activity and therefore muscle injuries [57]. It will be important to test newer, more potent SIRT1 activators in mdx mice. Interestingly, another study in a zebrafish model of muscular dystrophy showed that treatment with NAD^+^ or precursors improved the dystrophic phenotype [58]. Our data, along with these observations, suggest that treatment with potent and specific activators of SIRT1 can offer new therapeutic approaches for treating muscular dystrophies, and perhaps sarcopenia.

## Materials and Methods

### Animals

All animal procedures were performed according to Massachusetts Institute of Technology Committee on Animal Care. Mice were fed standard rodent chow diet and housed in a facility with 12 hr light and dark cycles. To generate muscle-specific transgenic mice (TG), the mouse *SIRT1* cDNA was cloned between the 4.8 kb promoter of muscle creatine kinase (*MCK*) [30] and the human growth hormone (*hGH*) polyadenyltion sequence. Mouse oocytes of the C57BL/6J genetic background were injected with this construct by the MIT transgenic facility. The SIRT1 muscle-specific knockout mice (MckKO) were generated by crossing mice being homozygous for the floxed *SIRT1* allele [42] with mice expressing the cre recombinase under the control of *MCK* promoter [41]. Both strains were in C57BL/6J genetic background. The mdx mice (C57BL/10ScSn-Dmd^mdx^/J) [45] were purchased from the Jackson Laboratories, and were crossed to SIRT1 Tg or MckKO mice to generate mdx;TG and mdx;MckKO mice. To control for variations in the genetic backgrounds all experiments were performed comparing siblings of same gender (either mdx *vs.* mdx;Tg or mdx;F/F *vs.* mdx;cre;F/F).

### Treadmill exercise

The treadmill exercise was performed using a motor-driven treadmill (Columbus Instruments). The running protocol used for Tg and MckKO mice, following 3 days of acclimatization at 15 m/min, was 5 min at 13 m/min (warm up) and then the speed was increased 1 m/min every minute up to 18 m/min and kept constant for 30 min. After 30 min, the speed was increased 1 m/min. The mice were considered exhausted and removed when they sat on the shocker for more than 20 sec. The running protocol we used for mdx mice was milder to allow the mice to run. Following 3 days of acclimatization at 8 m/min for 10 min, the mice ran at 4 m/min for 5 min (warm up) and the speed was increased 1 m/min every min up to 9 m/min. The mice were considered exhausted when they sat for more than 20 sec.

### Blood measurements

Plasma insulin levels were measured after overnight fasting using ELISA kit (Millipore). Blood glucose levels were measured using OneTouch strips and glucometer. To measure serum creatine kinase (CK) levels, the blood was collected in heparinized tubes, serum was isolated, and CK activity was assayed using the DiscretPak Creatinine Kinase Reagent Kit (Catachem) according to manufacturer's protocol.

### Histological analyses and Evans Blue staining

Gastrocnemius muscle was fixed with formaldehyde, paraffin-embedded, cross-sectioned and stained with hematoxylin and eosin following standard procedures. The slides were analyzed using standard light microscopy. The fibers' sizes (25–30/image) were counted in 10 randomly chosen images/mouse (250–300 fibers/mouse) using ImageJ software (NIH). For SDH, COX, and ATPase stainings, gastrocnemius/soleus muscles were snap frozen in isopentane/liquid N_2_, cryo-sectioned, and stained for enzymatic activities using standard procedures. The stained fibers were counted and their percentage of total number of fibers was calculated (150–200 total fibers/image, 5 images/mouse, 3 mice/genotype). Evans Blue dye (1% solution) was injected intraperitoneally (1% volume/gr of body weight), and the mice were euthanized 16 hr later. Gastrocnemius muscle was dissected and embedded in OCT compound, frozen, and cross-sectioned. The slides were also stained with DAPI to visualize nuclei. Evans Blue and DAPI staining were analyzed by fluorescence microscopy. The area of Evans Blue stained fibers was counted in 10 randomly chosen images/mouse by ImageJ and the percentage of total area was calculated.

### RNA, mitochondrial DNA, protein analyses, NAD^+^ measurements

RNA was isolated from gastrocnemius muscle using Trizol (Invitrogen) and further purified using RNeasy mini columns (Qiagen). Quantitative PCR analysis was performed on a LightCycler 480II (Roche) using iQ SYBR Green Supermix (Biorad). For mitochondrial DNA quantitation, mitochondrial and genomic DNA was isolated from gastrocnemius muscle after Proteinase K and RNAse A digestion followed by phenol-chloroform extraction. Quantitative PCR analyses were performed using mitochondrial and genomic specific primers.

Skeletal muscle protein homogenates were prepared following standard procedures. The antibodies used were against SIRT1 N-term (Millipore, #07-131), actin (Chemicon, MAB1501), AMPKα (Cell Signaling, #2603), phospho-AMPKα (Cell Signaling, #2531), p70 S6 Kinase (Cell Signaling, #2708), phospho-p70 S6 Kinase (Cell Signaling, #9205), Gapdh (Sigma, G9545), PGC1 (Santa Cruz, sc-13067), acetylated lysine (ImmuneChem, ICP0380). The immunoprecipitation was performed using the Pierce Direct-IP Kit (Thermo Scientific) according to manufacturer's instructions.

NAD^+^ was measured in freshly isolated gastrocnemius muscle using EnzyChrom kit from BioAssay Systems following the manufacturer's protocol.

## Supporting Information

Figure S1 SIRT1 transgenic lines. (A) Relative protein levels of SIRT1 in gastrocnemius muscle of WT and Tg-4140 line quantified by Image J (n = 4). SIRT1 protein is expressed at approximately ∼100 fold in Tg-4140 muscle compared to WT. (B) Western blot in tissue protein homogenates prepared from WT and SIRT1 transgenic lines 4311 and 4145. (He: heart, Qu: Quadriceps, Ga: gastrocnemius, So: soleus, L: liver.) (C) Gastrocnemius muscle weight (from one hindlimb) and muscle/body weight ratio of Tg-4311 and Tg-4145 mice at 8–10 weeks of age (n = 4–6). (D–G) Relative mRNA levels of cytochrome C, *TFAM*, *PGC-1α*, and *UCP3* in gastrocnemius muscle of WT, SIRT1 Tg-4311, and Tg-4145. Data are expressed as mean +/− s.e.m. *p<0.05, **p<0.01, ***p<0.001 by two-tailed unpaired Student's t test.
(PDF)

Figure S2 SIRT1 overexpression drives fast-to-slow fiber type switch. (A) Quantitation of fibers stained for SDH activity in WT and Tg-4140 muscle shown in **Figure 3C** (n = 3 animals, 500–1000 fibers counted/animal). (B) Quantitation of fibers stained for COX activity in WT and Tg-4140 muscle shown in **Figure 3D** (n = 3 animals, 500–1000 total fibers counted/animal). (C) Representative myosin ATPase activity staining at indicated pH of cross-sections of gastrocnemius muscle of WT and Tg-4140 mice (10× magnification). Type I fibers are stained light in pH 10.2, and dark in pH 4.31. Type II fibers are stained dark in pH 10.2 and light in pH 4.31 (n = 3 animals, 500–1000 fibers counted/animal).
(PDF)

Figure S3 Loss of SIRT1 from skeletal muscle does not affect the expression of atrophy genes under basal or atrophy-inducing conditions. (A) Relative mRNA levels of *MAFBx* and *MuRF-1* atrophy genes in gastrocnemius muscle of WT and MckKO mice fed or fasted for 24 hrs (n = 3–5). (B) Relative mRNA levels of *MAFBx* and *MuRF-1* atrophy genes in gastrocnemius muscle of WT and MckKO mice, which underwent mock surgery (control) or were denervated for 3 days (n = 3–5). Data are expressed as mean +/− s.e.m.
(PDF)

Figure S4 SIRT1 Tg-4145 exhibits protective signs against DMD, and SIRT1 loss from skeletal muscle does not affect the muscular dystrophic phenotype of mdx mouse. (A) Relative mRNA levels of utrophin in gastrocnemius muscle of Tg-4145 line (8–10 weeks old, n = 3) (B) Serum creatine kinase activity in mdx, mdx Tg-4145 mice (14 weeks old, n = 6–10). (C) Serum creatine kinase activity in mdx and mdx;MckKO mice (8–10 weeks old, n = 10). (D) Distance run in treadmill exercise by mdx and mdx;MckKO mice (12–14 weeks old, n = 10–12). (E) Relative mRNA levels of acetylcholine receptor (AchR) subunits α, δ, ε, *Musk*, *Erbb*, and *Raspn* in gastrocnemius muscle from WT and MckKO mice (n = 3–5), and (F) mdx and mdx;MckKO mice (n = 3–5). Data are expressed as mean +/− s.e.m. *p<0.05, n.s: non-significant.
(PDF)

## References

[1] Bassel-Duby R, Olson EN (2006) Signaling pathways in skeletal muscle remodeling. Annu Rev Biochem 75: 19–37. 16756483 10.1146/annurev.biochem.75.103004.142622

[2] Booth FW, Thomason DB (1991) Molecular and cellular adaptation of muscle in response to exercise: perspectives of various models. Physiol Rev 71: 541–585. 2006222 10.1152/physrev.1991.71.2.541

[3] Pette D, Staron RS (2001) Transitions of muscle fiber phenotypic profiles. Histochem Cell Biol 115: 359–372. 11449884 10.1007/s004180100268

[4] Berchtold MW, Brinkmeier H, Muntener M (2000) Calcium ion in skeletal muscle: its crucial role for muscle function, plasticity, and disease. Physiol Rev 80: 1215–1265. 10893434 10.1152/physrev.2000.80.3.1215

[5] Olson EN, Williams RS (2000) Remodeling muscles with calcineurin. Bioessays 22: 510–519. 11056484 10.1002/1521-1878(200011)22:11<1049::AID-BIES14>3.0.CO;2-M

[6] Aspnes LE, Lee CM, Weindruch R, Chung SS, Roecker EB, et al (1997) Caloric restriction reduces fiber loss and mitochondrial abnormalities in aged rat muscle. Faseb J 11: 573–581. 9212081 10.1096/fasebj.11.7.9212081

[7] Civitarese AE, Carling S, Heilbronn LK, Hulver MH, Ukropcova B, et al (2007) Calorie restriction increases muscle mitochondrial biogenesis in healthy humans. PLoS Med 4: e76. 17341128 10.1371/journal.pmed.0040076 PMC1808482

[8] Chalkiadaki A, Guarente L (2012) Sirtuins mediate mammalian metabolic responses to nutrient availability. Nat Rev Endocrinol 8: 287–296. 22249520 10.1038/nrendo.2011.225

[9] Chalkiadaki A, Guarente L (2012) High-fat diet triggers inflammation-induced cleavage of SIRT1 in adipose tissue to promote metabolic dysfunction. Cell Metab 16: 180–188. 22883230 10.1016/j.cmet.2012.07.003 PMC3539750

[10] Canto C, Gerhart-Hines Z, Feige JN, Lagouge M, Noriega L, et al (2009) AMPK regulates energy expenditure by modulating NAD+ metabolism and SIRT1 activity. Nature 458: 1056–1060. 19262508 10.1038/nature07813 PMC3616311

[11] Canto C, Jiang LQ, Deshmukh AS, Mataki C, Coste A, et al (2010) Interdependence of AMPK and SIRT1 for metabolic adaptation to fasting and exercise in skeletal muscle. Cell Metab 11: 213–219. 20197054 10.1016/j.cmet.2010.02.006 PMC3616265

[12] Gerhart-Hines Z, Rodgers JT, Bare O, Lerin C, Kim SH, et al (2007) Metabolic control of muscle mitochondrial function and fatty acid oxidation through SIRT1/PGC-1alpha. Embo J 26: 1913–1923. 17347648 10.1038/sj.emboj.7601633 PMC1847661

[13] Wu Z, Puigserver P, Andersson U, Zhang C, Adelmant G, et al (1999) Mechanisms controlling mitochondrial biogenesis and respiration through the thermogenic coactivator PGC-1. Cell 98: 115–124. 10412986 10.1016/S0092-8674(00)80611-X

[14] Lin J, Wu H, Tarr PT, Zhang CY, Wu Z, et al (2002) Transcriptional co-activator PGC-1 alpha drives the formation of slow-twitch muscle fibres. Nature 418: 797–801. 12181572 10.1038/nature00904

[15] Sandri M, Lin J, Handschin C, Yang W, Arany ZP, et al (2006) PGC-1alpha protects skeletal muscle from atrophy by suppressing FoxO3 action and atrophy-specific gene transcription. Proc Natl Acad Sci U S A 103: 16260–16265. 17053067 10.1073/pnas.0607795103 PMC1637570

[16] Wenz T, Rossi SG, Rotundo RL, Spiegelman BM, Moraes CT (2009) Increased muscle PGC-1alpha expression protects from sarcopenia and metabolic disease during aging. Proc Natl Acad Sci U S A 106: 20405–20410. 19918075 10.1073/pnas.0911570106 PMC2787152

[17] Choi CS, Befroy DE, Codella R, Kim S, Reznick RM, et al (2008) Paradoxical effects of increased expression of PGC-1alpha on muscle mitochondrial function and insulin-stimulated muscle glucose metabolism. Proc Natl Acad Sci U S A 105: 19926–19931. 19066218 10.1073/pnas.0810339105 PMC2598730

[18] Gomes AP, Price NL, Ling AJ, Moslehi JJ, Montgomery MK, et al (2013) Declining NAD(+) Induces a Pseudohypoxic State Disrupting Nuclear-Mitochondrial Communication during Aging. Cell 155: 1624–1638. 24360282 10.1016/j.cell.2013.11.037 PMC4076149

[19] Handschin C, Kobayashi YM, Chin S, Seale P, Campbell KP, et al (2007) PGC-1alpha regulates the neuromuscular junction program and ameliorates Duchenne muscular dystrophy. Genes Dev 21: 770–783. 17403779 10.1101/gad.1525107 PMC1838529

[20] Bogdanovich S, Perkins KJ, Krag TO, Khurana TS (2004) Therapeutics for Duchenne muscular dystrophy: current approaches and future directions. J Mol Med 82: 102–115. 14673527 10.1007/s00109-003-0484-1

[21] Webster C, Silberstein L, Hays AP, Blau HM (1988) Fast muscle fibers are preferentially affected in Duchenne muscular dystrophy. Cell 52: 503–513. 3342447 10.1016/0092-8674(88)90463-1

[22] Ibraghimov-Beskrovnaya O, Ervasti JM, Leveille CJ, Slaughter CA, Sernett SW, et al (1992) Primary structure of dystrophin-associated glycoproteins linking dystrophin to the extracellular matrix. Nature 355: 696–702. 1741056 10.1038/355696a0

[23] Byers TJ, Kunkel LM, Watkins SC (1991) The subcellular distribution of dystrophin in mouse skeletal, cardiac, and smooth muscle. J Cell Biol 115: 411–421. 1918148 10.1083/jcb.115.2.411 PMC2289158

[24] Sealock R, Butler MH, Kramarcy NR, Gao KX, Murnane AA, et al (1991) Localization of dystrophin relative to acetylcholine receptor domains in electric tissue and adult and cultured skeletal muscle. J Cell Biol 113: 1133–1144. 2040646 10.1083/jcb.113.5.1133 PMC2289019

[25] Love DR, Hill DF, Dickson G, Spurr NK, Byth BC, et al (1989) An autosomal transcript in skeletal muscle with homology to dystrophin. Nature 339: 55–58. 2541343 10.1038/339055a0

[26] Miura P, Jasmin BJ (2006) Utrophin upregulation for treating Duchenne or Becker muscular dystrophy: how close are we? Trends Mol Med 12: 122–129. 16443393 10.1016/j.molmed.2006.01.002

[27] Tinsley J, Deconinck N, Fisher R, Kahn D, Phelps S, et al (1998) Expression of full-length utrophin prevents muscular dystrophy in mdx mice. Nat Med 4: 1441–1444. 9846586 10.1038/4033

[28] Tinsley JM, Potter AC, Phelps SR, Fisher R, Trickett JI, et al (1996) Amelioration of the dystrophic phenotype of mdx mice using a truncated utrophin transgene. Nature 384: 349–353. 8934518 10.1038/384349a0

[29] Selsby JT, Morine KJ, Pendrak K, Barton ER, Sweeney HL (2012) Rescue of dystrophic skeletal muscle by PGC-1alpha involves a fast to slow fiber type shift in the mdx mouse. PLoS One 7: e30063. 22253880 10.1371/journal.pone.0030063 PMC3256197

[30] Johnson JE, Wold BJ, Hauschka SD (1989) Muscle creatine kinase sequence elements regulating skeletal and cardiac muscle expression in transgenic mice. Mol Cell Biol 9: 3393–3399. 2796990 10.1128/mcb.9.8.3393-3399.1989 PMC362385

[31] Gomes MD, Lecker SH, Jagoe RT, Navon A, Goldberg AL (2001) Atrogin-1, a muscle-specific F-box protein highly expressed during muscle atrophy. Proc Natl Acad Sci U S A 98: 14440–14445. 11717410 10.1073/pnas.251541198 PMC64700

[32] Lecker SH, Jagoe RT, Gilbert A, Gomes M, Baracos V, et al (2004) Multiple types of skeletal muscle atrophy involve a common program of changes in gene expression. Faseb J 18: 39–51. 14718385 10.1096/fj.03-0610com

[33] Sandri M, Sandri C, Gilbert A, Skurk C, Calabria E, et al (2004) Foxo transcription factors induce the atrophy-related ubiquitin ligase atrogin-1 and cause skeletal muscle atrophy. Cell 117: 399–412. 15109499 10.1016/s0092-8674(04)00400-3 PMC3619734

[34] Stitt TN, Drujan D, Clarke BA, Panaro F, Timofeyva Y, et al (2004) The IGF-1/PI3K/Akt pathway prevents expression of muscle atrophy-induced ubiquitin ligases by inhibiting FOXO transcription factors. Mol Cell 14: 395–403. 15125842 10.1016/s1097-2765(04)00211-4

[35] Chen D, Bruno J, Easlon E, Lin SJ, Cheng HL, et al (2008) Tissue-specific regulation of SIRT1 by calorie restriction. Genes Dev 22: 1753–1757. 18550784 10.1101/gad.1650608 PMC2492662

[36] Schiaffino S, Reggiani C (1994) Myosin isoforms in mammalian skeletal muscle. J Appl Physiol 77: 493–501. 8002492 10.1152/jappl.1994.77.2.493

[37] Rodgers JT, Lerin C, Haas W, Gygi SP, Spiegelman BM, et al (2005) Nutrient control of glucose homeostasis through a complex of PGC-1alpha and SIRT1. Nature 434: 113–118. 15744310 10.1038/nature03354

[38] Hondares E, Pineda-Torra I, Iglesias R, Staels B, Villarroya F, et al (2007) PPARdelta, but not PPARalpha, activates PGC-1alpha gene transcription in muscle. Biochem Biophys Res Commun 354: 1021–1027. 17275789 10.1016/j.bbrc.2007.01.092

[39] Price NL, Gomes AP, Ling AJ, Duarte FV, Martin-Montalvo A, et al (2012) SIRT1 is required for AMPK activation and the beneficial effects of resveratrol on mitochondrial function. Cell Metab 15: 675–690. 22560220 10.1016/j.cmet.2012.04.003 PMC3545644

[40] Laplante M, Sabatini DM (2012) mTOR signaling in growth control and disease. Cell 149: 274–293. 22500797 10.1016/j.cell.2012.03.017 PMC3331679

[41] Bruning JC, Michael MD, Winnay JN, Hayashi T, Horsch D, et al (1998) A muscle-specific insulin receptor knockout exhibits features of the metabolic syndrome of NIDDM without altering glucose tolerance. Mol Cell 2: 559–569. 9844629 10.1016/s1097-2765(00)80155-0

[42] Cheng HL, Mostoslavsky R, Saito S, Manis JP, Gu Y, et al (2003) Developmental defects and p53 hyperacetylation in Sir2 homolog (SIRT1)-deficient mice. Proc Natl Acad Sci U S A 100: 10794–10799. 12960381 10.1073/pnas.1934713100 PMC196882

[43] Menzies KJ, Singh K, Saleem A, Hood DA (2013) Sirtuin 1-mediated effects of exercise and resveratrol on mitochondrial biogenesis. J Biol Chem 288: 6968–6979. 23329826 10.1074/jbc.M112.431155 PMC3591607

[44] Philp A, Chen A, Lan D, Meyer GA, Murphy AN, et al (2011) Sirtuin 1 (SIRT1) deacetylase activity is not required for mitochondrial biogenesis or peroxisome proliferator-activated receptor-gamma coactivator-1alpha (PGC-1alpha) deacetylation following endurance exercise. J Biol Chem 286: 30561–30570. 21757760 10.1074/jbc.M111.261685 PMC3162416

[45] Bulfield G, Siller WG, Wight PA, Moore KJ (1984) X chromosome-linked muscular dystrophy (mdx) in the mouse. Proc Natl Acad Sci U S A 81: 1189–1192. 6583703 10.1073/pnas.81.4.1189 PMC344791

[46] Ghedini PC, Viel TA, Honda L, Avellar MC, Godinho RO, et al (2008) Increased expression of acetylcholine receptors in the diaphragm muscle of MDX mice. Muscle Nerve 38: 1585–1594. 19016551 10.1002/mus.21183

[47] Brussee V, Tardif F, Tremblay JP (1997) Muscle fibers of mdx mice are more vulnerable to exercise than those of normal mice. Neuromuscul Disord 7: 487–492. 9447605 10.1016/s0960-8966(97)00115-6

[48] Schenk S, McCurdy CE, Philp A, Chen MZ, Holliday MJ, et al (2011) Sirt1 enhances skeletal muscle insulin sensitivity in mice during caloric restriction. J Clin Invest 121: 4281–4288. 21985785 10.1172/JCI58554 PMC3204844

[49] Guarente L (2011) Sirtuins, aging, and metabolism. Cold Spring Harb Symp Quant Biol 76: 81–90. 22114328 10.1101/sqb.2011.76.010629

[50] Arany Z, He H, Lin J, Hoyer K, Handschin C, et al (2005) Transcriptional coactivator PGC-1 alpha controls the energy state and contractile function of cardiac muscle. Cell Metab 1: 259–271. 16054070 10.1016/j.cmet.2005.03.002

[51] Handschin C, Chin S, Li P, Liu F, Maratos-Flier E, et al (2007) Skeletal muscle fiber-type switching, exercise intolerance, and myopathy in PGC-1alpha muscle-specific knock-out animals. J Biol Chem 282: 30014–30021. 17702743 10.1074/jbc.M704817200

[52] Geng T, Li P, Okutsu M, Yin X, Kwek J, et al (2010) PGC-1alpha plays a functional role in exercise-induced mitochondrial biogenesis and angiogenesis but not fiber-type transformation in mouse skeletal muscle. Am J Physiol Cell Physiol 298: C572–579. 20032509 10.1152/ajpcell.00481.2009 PMC3353735

[53] Rowe GC, El-Khoury R, Patten IS, Rustin P, Arany Z (2012) PGC-1alpha is dispensable for exercise-induced mitochondrial biogenesis in skeletal muscle. PLoS One 7: e41817. 22848618 10.1371/journal.pone.0041817 PMC3404101

[54] Cohen HY, Miller C, Bitterman KJ, Wall NR, Hekking B, et al (2004) Calorie restriction promotes mammalian cell survival by inducing the SIRT1 deacetylase. Science 305: 390–392. 15205477 10.1126/science.1099196

[55] Hubbard BP, Gomes AP, Dai H, Li J, Case AW, et al (2013) Evidence for a common mechanism of SIRT1 regulation by allosteric activators. Science 339: 1216–1219. 23471411 10.1126/science.1231097 PMC3799917

[56] Howitz KT, Bitterman KJ, Cohen HY, Lamming DW, Lavu S, et al (2003) Small molecule activators of sirtuins extend Saccharomyces cerevisiae lifespan. Nature 425: 191–196. 12939617 10.1038/nature01960

[57] Hori YS, Kuno A, Hosoda R, Tanno M, Miura T, et al (2011) Resveratrol ameliorates muscular pathology in the dystrophic mdx mouse, a model for Duchenne muscular dystrophy. J Pharmacol Exp Ther 338: 784–794. 21652783 10.1124/jpet.111.183210

[58] Goody MF, Kelly MW, Reynolds CJ, Khalil A, Crawford BD, et al (2012) NAD+ biosynthesis ameliorates a zebrafish model of muscular dystrophy. PLoS Biol 10: e1001409. 23109907 10.1371/journal.pbio.1001409 PMC3479101

