# Supplementary figures and images for: Muscle-Specific SIRT1 Gain-of-Function Increases Slow-Twitch Fibers and Ameliorates Pathophysiology in a Mouse Model of Duchenne Muscular Dystrophy
Source: PLoS Genet. 2014 Jul 17;10(7):e1004490. doi: 10.1371/journal.pgen.1004490 (PMC4102452; doi:10.1371/journal.pgen.1004490)

Supplementary Figure 1

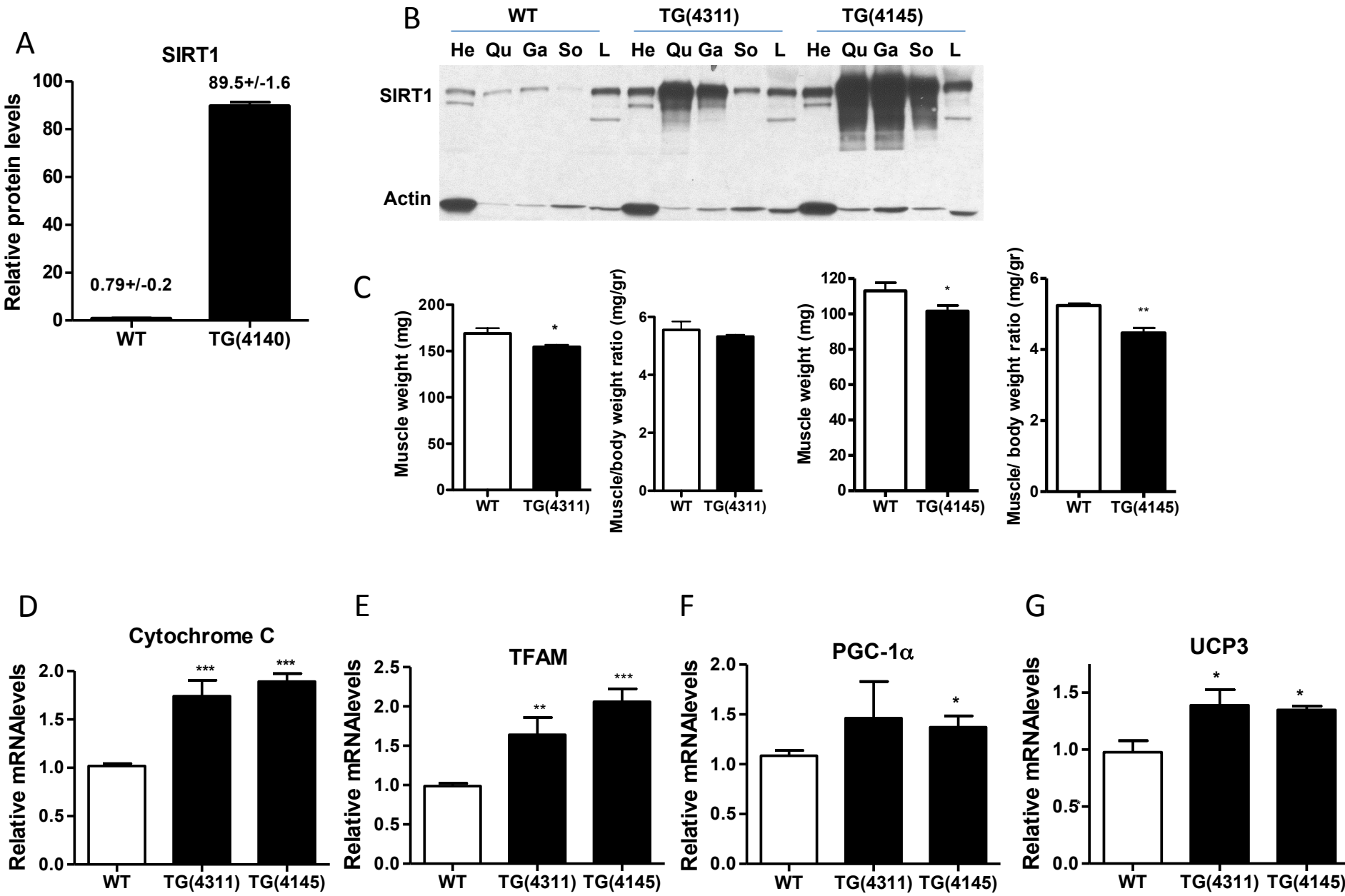

Supplement: Figure S1 — SIRT1 transgenic lines. (A) Relative protein levels of SIRT1 in gastrocnemius muscle of WT and Tg-4140 line quantified by Image J (n = 4). SIRT1 protein is expressed at approximately ∼100 fold in Tg-4140 muscle compared to WT. (B) Western blot in tissue protein homogenates prepared from WT and SIRT1 transgenic lines 4311 and 4145. (He: heart, Qu: Quadriceps, Ga: gastrocnemius, So: soleus, L: liver.) (C) Gastrocnemius muscle weight (from one hindlimb) and muscle/body weight ratio of Tg-4311 and Tg-4145 mice at 8–10 weeks of age (n = 4–6). (D–G) Relative mRNA levels of cytochrome C, TFAM, PGC-1α, and UCP3 in gastrocnemius muscle of WT, SIRT1 Tg-4311, and Tg-4145. Data are expressed as mean +/− s.e.m. *p<0.05, **p<0.01, ***p<0.001 by two-tailed unpaired Student's t test. (PDF) [file pgen.1004490.s001.pdf]

Supplementary Figure 2

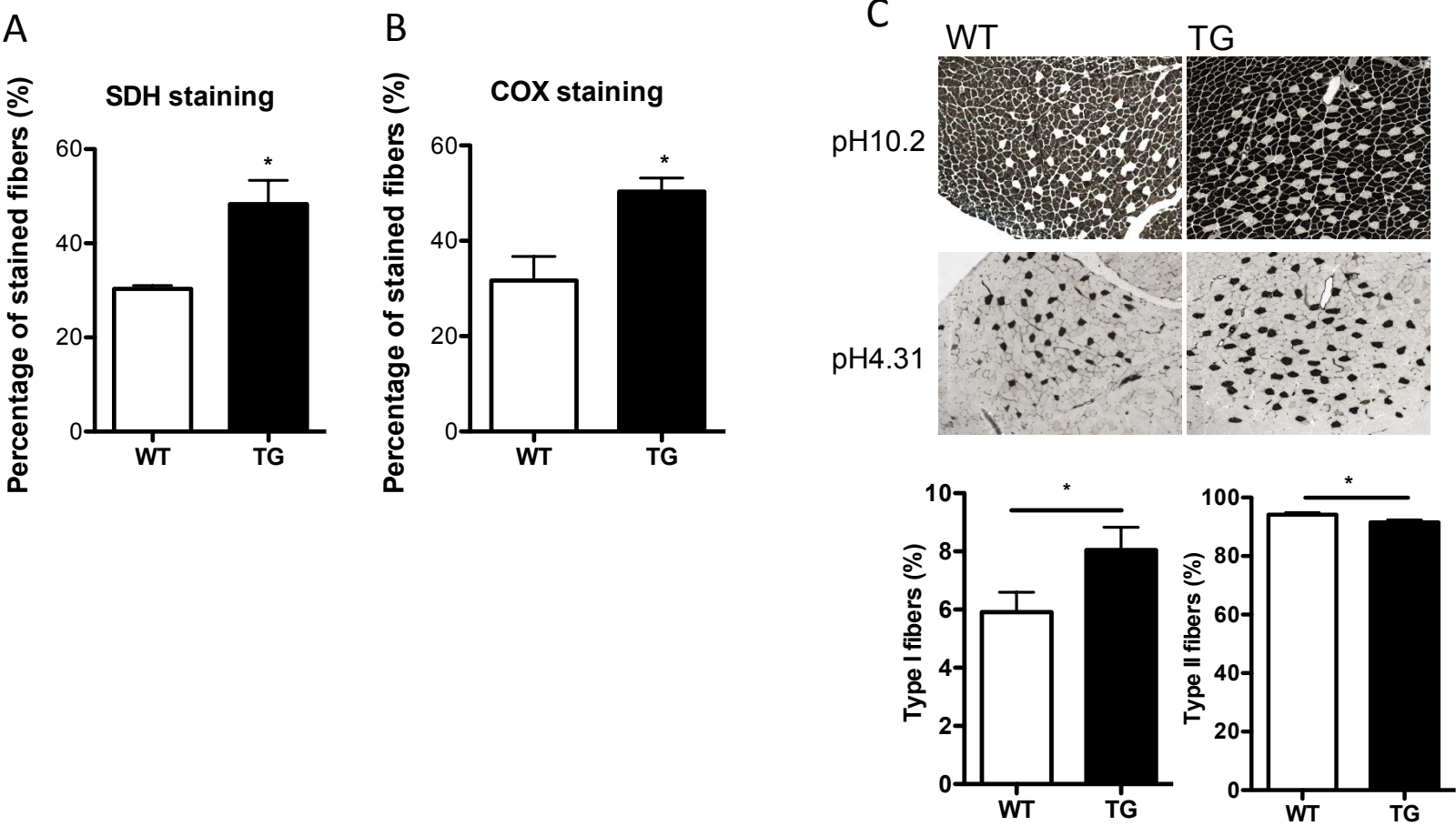

Supplement: Figure S2 — SIRT1 overexpression drives fast-to-slow fiber type switch. (A) Quantitation of fibers stained for SDH activity in WT and Tg-4140 muscle shown in Figure 3C (n = 3 animals, 500–1000 fibers counted/animal). (B) Quantitation of fibers stained for COX activity in WT and Tg-4140 muscle shown in Figure 3D (n = 3 animals, 500–1000 total fibers counted/animal). (C) Representative myosin ATPase activity staining at indicated pH of cross-sections of gastrocnemius muscle of WT and Tg-4140 mice (10× magnification). Type I fibers are stained light in pH 10.2, and dark in pH 4.31. Type II fibers are stained dark in pH 10.2 and light in pH 4.31 (n = 3 animals, 500–1000 fibers counted/animal). (PDF) [file pgen.1004490.s002.pdf]

Supplementary Figure 3

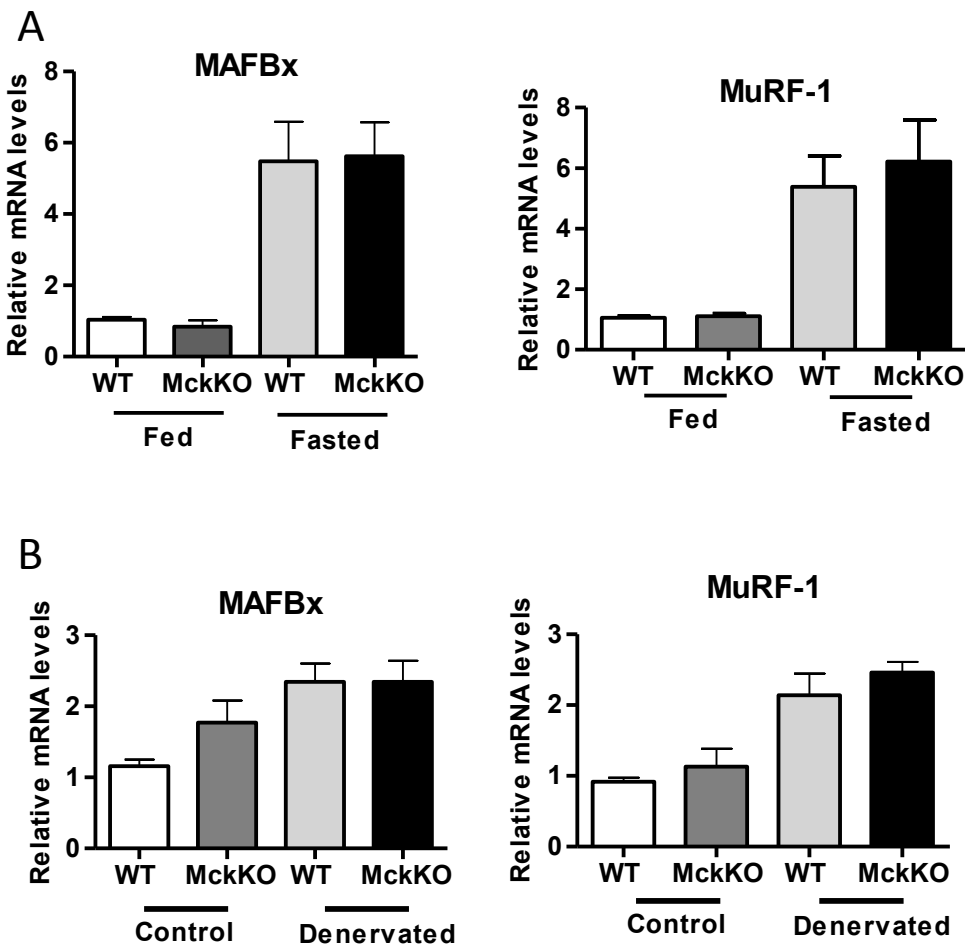

Supplement: Figure S3 — Loss of SIRT1 from skeletal muscle does not affect the expression of atrophy genes under basal or atrophy-inducing conditions. (A) Relative mRNA levels of MAFBx and MuRF-1 atrophy genes in gastrocnemius muscle of WT and MckKO mice fed or fasted for 24 hrs (n = 3–5). (B) Relative mRNA levels of MAFBx and MuRF-1 atrophy genes in gastrocnemius muscle of WT and MckKO mice, which underwent mock surgery (control) or were denervated for 3 days (n = 3–5). Data are expressed as mean +/− s.e.m. (PDF) [file pgen.1004490.s003.pdf]

Supplementary Figure 4

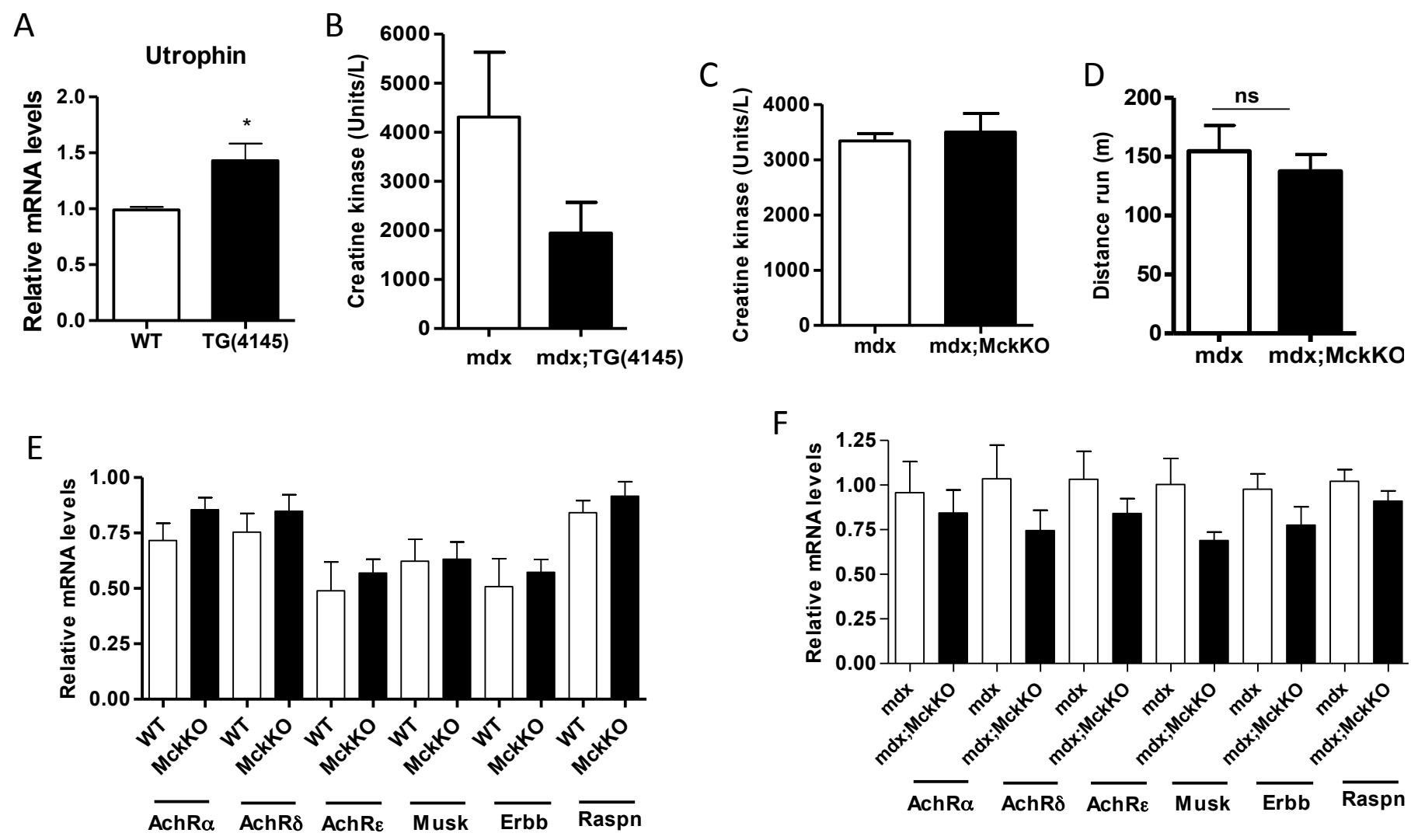

Supplement: Figure S4 — SIRT1 Tg-4145 exhibits protective signs against DMD, and SIRT1 loss from skeletal muscle does not affect the muscular dystrophic phenotype of mdx mouse. (A) Relative mRNA levels of utrophin in gastrocnemius muscle of Tg-4145 line (8–10 weeks old, n = 3) (B) Serum creatine kinase activity in mdx, mdx Tg-4145 mice (14 weeks old, n = 6–10). (C) Serum creatine kinase activity in mdx and mdx;MckKO mice (8–10 weeks old, n = 10). (D) Distance run in treadmill exercise by mdx and mdx;MckKO mice (12–14 weeks old, n = 10–12). (E) Relative mRNA levels of acetylcholine receptor (AchR) subunits α, δ, ε, Musk, Erbb, and Raspn in gastrocnemius muscle from WT and MckKO mice (n = 3–5), and (F) mdx and mdx;MckKO mice (n = 3–5). Data are expressed as mean +/− s.e.m. *p<0.05, n.s: non-significant. (PDF) [file pgen.1004490.s004.pdf]
